# A mobile genetic element with unknown function found in distantly related viruses

**DOI:** 10.1186/1743-422X-10-132

**Published:** 2013-04-25

**Authors:** Torstein Tengs, Anja Bråthen Kristoffersen, Tsvetan R Bachvaroff, Christine Monceyron Jonassen

**Affiliations:** 1 National Veterinary Institute, P.O. Box 750 Sentrum, 0106, Oslo, Norway; 2 Institute for Marine and Environmental Technology, 701 E. Pratt St, Baltimore, MD 21202, USA

**Keywords:** Astroviruses, Caliciviruses, Picornaviruses, Coronaviruses, Mobile genetic element, s2m

## Abstract

**Background:**

The genetic element s2m seems to represent one of very few examples of mobile genetic elements in viruses. The function remains obscure and a scattered taxonomical distribution has been reported by numerous groups.

**Methods:**

We have searched GenBank in order to identify all viral accessions that have s2m(−like) sequence motifs. Rigorous phylogenetic analyses and constrained tree topology testing were also performed in order to investigate the apparently mobile nature of s2m.

**Results:**

The stem-loop s2m structure can be found in four families of + ssRNA viruses; *Astroviridae*, *Caliciviridae*, *Picornaviridae* and *Coronaviridae*. In all of these virus families, with the possible exception of *Caliciviridae*, multiple gains and/or losses of s2m would have to be postulated in order to explain the distribution of this character.

**Conclusions:**

s2m appears to be a mobile genetic element with a unique evolutionary history in all of the four virus families where it can be found. Based on our findings and a review of the current literature on s2m, a hypothesis implying an RNAi-like function for the s2m element is also outlined.

## Background

In 1997, a 43 basepair, conserved sequence motif was described near the 3’ end of members of the *Astroviridae* family [1]. The genetic element corresponded to the second most 3’ stem-loop structure (stem-loop II) in human astroviruses and was subsequently named s2m [2]. The sequence motif has later been found in three other virus families; *Caliciviridae*, *Picornaviridae* and *Coronaviridae*[3]. The distribution of s2m seems to be limited to positive-sense, single-stranded RNA (+ssRNA) viruses and the element is always located near the 3’ end of the genome. Most commonly, s2m can be found downstream of the last reading frame, but there are also instances where it appears that the final stop codon is part of the motif itself [2]. Recently, several examples of viruses containing two copies of s2m have also been reported [3]. The level of conservation is particularly striking given the high mutation rates seen in RNA viruses.

The s2m sequence is also present in the genome of the severe acute respiratory syndrome (SARS) coronavirus (SARS-CoV) where its three-dimensional crystal structure has been characterized in great detail [4]. The function of s2m remains obscure, although in the case of SARS-CoV, it was suggested that the structure might interfere with protein synthesis through mimicry of small subunit ribosomal RNA (SSU rRNA) and subsequent binding of ribosomal proteins [4]. This proposed affinity was, however, only observed when interactions were modeled using prokaryotic proteins. One of the many interesting features of s2m is that there seems to be a high level of conservation on all levels; primary structure (sequence) of both stem and loop regions, secondary (stem/loop structure) and tertiary conformation, indicating that all of these characteristics are important for functionality. The conserved nature of s2m has led researchers to suggest using it as a drug target [4] and there are also protocols describing how primers targeting the conserved s2m sequence can be used for virus discovery using reverse transcription PCR [5].

The phylogenetic distribution of s2m seems to support a model where the genetic element can be transferred horizontally and mobility of s2m has been suggested in the literature [3]. We present a thorough analysis of the distribution of s2m in viral genomes and perform likelihood-based phylogenetic analyses of all the relevant virus groups. We propose models for the evolutionary history of s2m within the different host virus families and perform likelihood ratio tests to investigate the apparent mobile nature of this sequence motif.

## Results

A total of 682 s2m-containing sequences were found in GenBank when a consensus sequence-based approach was used directly. Allowing a single nucleotide mismatch increased this number to 702 and when two mismatches were allowed, a total of 706 sequences could be found from four different virus families (Additional file 1). Within this set of accessions, representatives from all the s2m-containing virus families were found that contained two copies of s2m (Table 1), but no sequences were found to contain three or more. The s2m sequences from genomes with two copies were never identical to each other. The genomes of dog norovirus strains GVI.1/HKU_Ca026F/2007/HKG and GVI.1/HKU_Ca035F/2007/HKG had identical 3’ends, both with two copies of s2m, although there were minor sequence differences in the rest of the sequences.

**Table 1:** GenBank accessions containing two copies of s2m

**Family**	**Virus (strain/isolate)**	**Accession number**
*Caliciviridae*	Norovirus dog (GVI.1/HKU_Ca026F/2007/HKG)*	FJ692500.1
*Caliciviridae*	Norovirus dog (GVI.1/HKU_Ca035F/2007/HKG)*	FJ692501.1
*Picornaviridae*	Pigeon picornavirus (B 03/641)	FR727144.1
*Picornaviridae*	Pigeon picornavirus (A 03/603)	FR727145.1
*Coronaviridae*	Zaria bat coronavirus (ZBCoV)	HQ166910.1
*Astroviridae*	Chicken astrovirus (FP3)	JN582328.1
*Astroviridae*	Chicken astrovirus (11672)	JN582327.1
*Astroviridae*	Chicken astrovirus (GA2011)	JF414802.1
*Astroviridae*	Bat astrovirus (Tm/Guangxi/LD77/2007)	FJ571066.1
*Astroviridae*	HMO Astrovirus (B NI-196)**	GQ415661.1

* - identical 3’ ends (including s2m motifs).

** - possibly due to incorrect sequence assembly.

**Figure 1:**
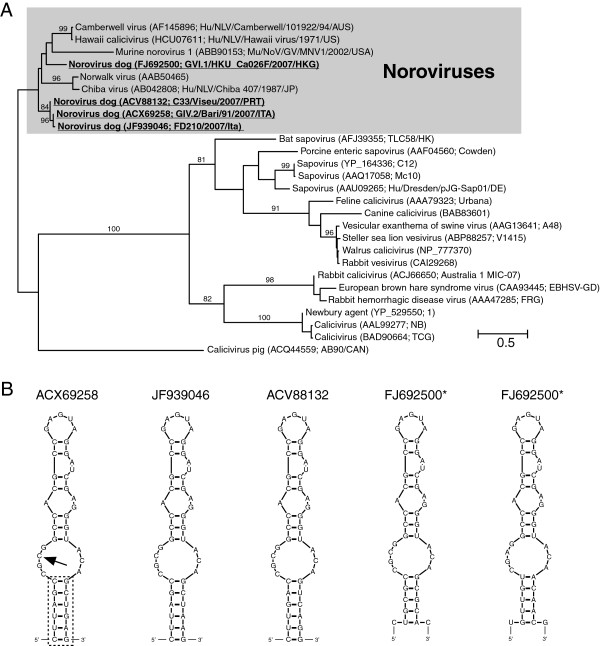
**s2m in caliciviruses. (A)** Phylogenetic analysis of caliciviruses using partial RNA-dependent RNA polymerase sequences (265 amino acids) and maximum likelihood (RAxML). Bootstrap values > 80% have been indicated and s2m-containing sequences have been highlighted (boldface/underscore). **(B)** Secondary structure of norovirus s2m sequences folded using mfold [6]. Box and arrow indicate nucleotide positions that vary within the noroviruses. * - The two copies of s2m found in Norovirus dog (GVI.1/HKU_Ca026F/2007/HKG) and Norovirus dog (GVI.1/HKU_Ca035F/2007/HKG).

**Figure 2:**
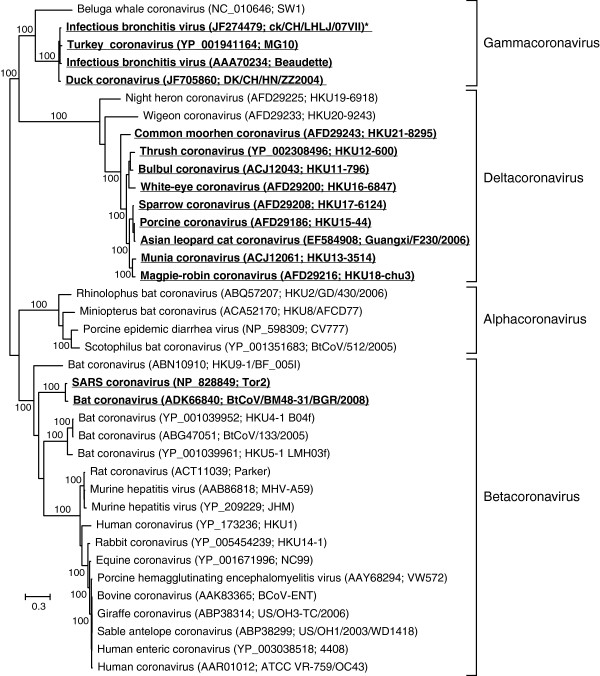
**Phylogenetic analysis of coronaviruses using partial polyprotein sequences (2833 amino acids) and maximum likelihood (RAxML).** Bootstrap values of 100% have been indicated and s2m-containing sequences have been highlighted (boldface/underscore).

**Figure 3:**
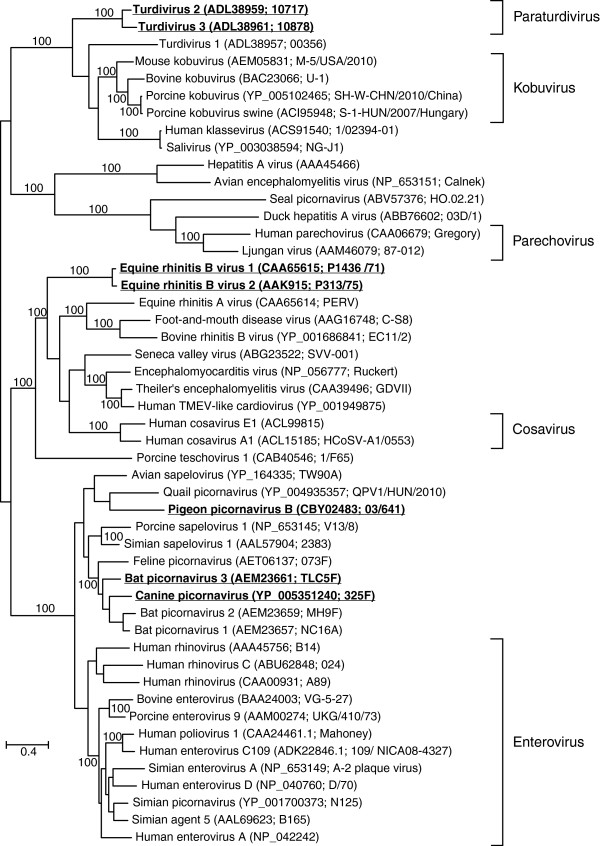
**Phylogenetic analysis of picornaviruses using partial polyprotein sequences (998 amino acids) and maximum likelihood (RAxML).** Bootstrap values of 100% have been indicated and s2m-containing sequences have been highlighted (boldface/underscore).

**Figure 4:**
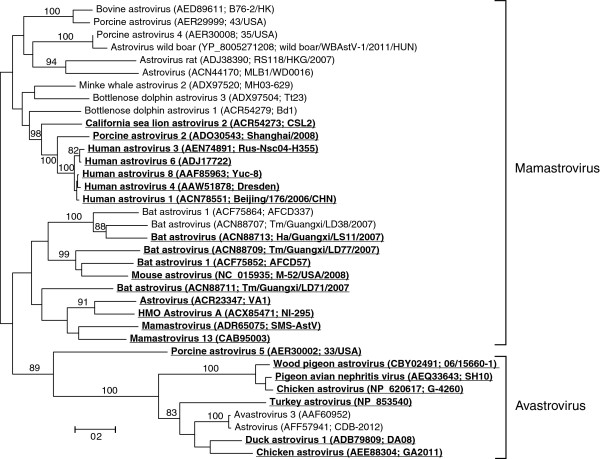
**Phylogenetic analysis of astroviruses using partial RNA-dependent RNA polymerase sequences (257 amino acids) and maximum likelihood (RAxML).** Bootstrap values > 80% have been indicated and s2m-containing sequences have been highlighted (boldface/underscore).

For the astroviruses and caliciviruses, partial RNA-dependent RNA polymerase (*RdRP*) amino acid sequences were used for the phylogenetic analyses (257 and 265 amino acid residues, respectively), whereas larger parts of the polyprotein sequence could be unambiguously aligned for the picornaviruses (998 residues) and the coronaviruses (2833 residues). The s2m-containing viruses did not form monophyletic groups in any of the four virus families (Figures 1A, 2, 3 and 4). Except for the coronaviruses, numerous sequences were obtained from isolates that had not been assigned a specific taxonomic placement within their respective families in the NCBI Taxonomy database, but in general, all trees constructed were consistent with previously published reports on viral phylogeny.

The ln likelihood difference (Δln) between unconstrained optimal trees and the optimal tree where all s2m sequences were required to be holophyletic (i.e. a single origin of the s2m sequence) ranged from coronaviruses (3102.9), picornaviruses (939.0), astroviruses (392.3), to caliciviruses (3.4). In all but the case of the caliciviruses, monophyly of the s2m-containing viruses was rejected with p < 0.01 (Additional file 2: Figure S1). Furthermore, even constrained trees assuming two gains of s2m were strongly rejected for coronaviruses, picornaviruses and astroviruses (Additional file 2: Figure S2, S3 and S4). For example in the picornaviruses one explanation for the distribution of the s2m sequence could be that the sequence was gained four times (Figure 3). The Approximately Unbiased (AU) test found that the tree where the s2m sequences were constrained into a single clade (requiring holophyly of s2m containing taxa) was significantly worse than the optimal tree (Additional file 2: Figure S3). Even constraining Bat picornavirus 3 and Canine picornavirus into a single clade was significantly worse than the optimal tree (Additional file 2: Figure S3).

## Discussion

Traditionally, the term mobile genetic elements (MGEs) has been restricted to include bacteriophages, plasmids and transposons, although it is now widely recognized that this classification is becoming obsolete as many elements with novel features as well as new combinations of known features are found [7,8]. Homologous and non-homologous recombination events have been inferred based on observations done in a wide range of virus groups, particularly in the single-stranded RNA viruses [9]. The genomes of bacteriophages are also known to contain genetic material from multiple sources and appear to be quite promiscuous when it comes to acquiring novel genomic features [10]. For non-phage viruses, mobile genetic elements seem very rare. To our knowledge, the only other example of what appears to be a mobile genetic element in regular viruses is the S7 domain found in certain double-stranded RNA viruses, although the degree of amino acid conservation is quite low [11].

No highly supported branches separated the dog norovirus sequences in the calicivirus group (Figure 1A), so a parsimonious model for the distribution of s2m here would be a single gain of this character. In contrast to what was found when investigating the other three s2m-containing virus families, a single gain of s2m was not rejected by the AU test. Phylogenetic analyses of the s2m sequences themselves (Figure 1B) could not, however, resolve the evolutionary history of s2m for this group, most likely due to residues either being too rapidly evolving or too conserved to give a good phylogenetic signal (data not shown). For the coronaviruses, two gains and two losses would have to be postulated in order to explain the distribution of s2m in the most parsimonious way (Figure 2). If the ancestral state for the gamma/delta coronavirus group was to contain s2m, losses would have to be proposed for the Night heron and Wigeon isolates, albeit there was little support separating these species so they could represent a single loss. Loss would have to be proposed, however, for the Beluga whale coronavirus. The s2m-containing, monophyletic group comprising the SARS virus and a bat coronavirus can be explained through a single gain. In the picornaviruses, there appears to a more complex distribution of s2m (Figure 3). Single gains can explain the two monophyletic groups that have s2m (paraturdiviruses and Equine rhinitis virus B 1 and 2) and the presence of s2m in Pigeon picornavirus B, but the phylogenetic placement of the two other s2m viruses (Bat picornavirus 3 and Canine picornavirus) is more ambiguous. They are not separated by highly supported branches and could thus reflect a single gain, or more complex explanations can be proposed, implying multiple gains and losses.

The broad distribution of s2m in the astroviruses (Figure 4) has been noticed previously [3] and a possible explanation could be that the ancestral state for this entire family was to contain s2m. For this to be true, a single loss would have to be proposed for the avastroviruses (Avastrovirus 3 and Astrovirus strain CDB-2012). Two losses would have to have occurred in Bat astrovirus 1 and Bat astrovirus strain Tm/Guangxi/LD38/2007. All members of the large, monophyletic group that includes the classical human astroviruses and has the California sea lion astrovirus 2 as the most basal branch contain s2m. This group is not separated from the rest of the (non-s2m containing) mamastroviruses by any highly supported branches, and is thus possible that this is a ‘primitive’ member of this virus family and that the absence of s2m in the remaining isolates can be explained through a single loss.

It is intuitive that loss of a complex character, and in particular a character that can only provide an evolutionary advantage in a direct or indirect interplay with an existing cellular mechanism and does not lead to a tremendous increase in fitness, is more likely than gain of such a feature in an evolutionary perspective. Given the data currently available for s2m we conclude that it is impossible to establish a statistical model that can take into account any such differences. In spite of this, we still believe that horizontal transfer is the most plausible explanation for the distribution of s2m. It is also formally possible that this is a case of convergent evolution, but we consider this highly unlikely given the high degree of s2m similarity and the complexity of the character. Alternative hypotheses would have to propose that s2m was present in the last common ancestor of the + ssRNA viruses. There is also an apparent lack of intermediate/primitive forms of s2m motifs in GenBank. It is probable that our search strategy for s2m(−like) motifs has a certain false negative rate, but the number of sequences that could be found quickly reached a plateau, where allowing more substitutions or fixing a smaller number of consensus motif characters only led to an exponential increase in the number of obvious false positives (data not shown).

A model where containing the s2m motif is the proposed ancestral state for all + ssRNA viruses would have to postulate a large number of independent losses and a extraordinary selection pressure to maintain s2m in certain viral lineages. This seems unlikely as s2m only appears to provide a somewhat subtle (yet immediate) selective advantage for the host viruses. The fact that s2m remains conserved in spite of the high mutation rates seen in RNA viruses indicates that the virus somehow benefits from acquiring the sequence motif, but unfortunately there are very few examples from the published literature on closely related viruses that differ in their s2m status. The turdiviruses (Figure 3) were all collected from dead birds, and the authors were unsuccessful in their efforts to culture the viral strains for further characterization [12]. All bats that were found to contain bat picornaviruses appeared healthy (Figure 3 and [13]) and there is no data indicating that the two viral strains that apparently have lost s2m within the delta/gammacoronavirus group (Night-heron coronavirus strain HKU19-6918 and Wigeon coronavirus strain HKU20-9243) are significantly different from the other members of this group in terms of pathogenicity, host specificity, etc. [14]. Exchanging the (non-s2m containing) 3’-end of a murine coronavirus (MCV) with the (s2m containing) 3’-end of a SARS-CoV did not appear to have a dramatic effect on the virus [15] and an IBV strain with a deleted version of s2m that was discovered as an escape mutant in a vaccine development project (GenBank accession number JF274479) did not appear phenotypically different from closely related viruses in culturing experiments (Dr. Shengwang Liu, personal communication).

The viruses that contain s2m can infect a wide range of higher vertebrates, including birds, bats, horses, dogs and humans, and display different tissue tropisms. The most likely scenario for the emergence of a new s2m- containing virus would be a situation where a co-infection includes both an s2m-containing donor virus and a recipient virus. Bats have been shown to carry many different viruses, including members of all the families that have been shown to harbor s2m [13,16]. Due to their mobility, feeding habits, long life span, roosting behavior, general virus susceptibility, etc., it has been proposed that bats may represent an important reservoir for emerging viruses [17]. However, for the coronaviruses, several s2m-containg members have been postulated to have an avian origin [14]. On a molecular level, is has been suggested that transfer of s2m occurs through non-homologous recombination in an replication-dependent manner [3]. Based on our sequence alignments, it is impossible to determine whether or not such a model should include just the hairpin structure or if transfer of the entire 3’ end of the genome represents a more likely scenario. The non-coding nature of this part of the genome is associated with high mutation rates and any sequence similarity in the s2m-flanking regions (particularly downstream, near the poly(A) tail) would quickly be lost due to the high error rates observed in replicating RNA virus genomes [18].

In most cases where phylogenetic analyses are used to investigate the horizontal transfer of a genetic element or a DNA-containing organelle such as plastids or mitochondria, it is possible to do a phylogenetic analysis of the genetic element that has (presumably) been transferred and then compare the resulting topology with that of the hosts. s2m is short, but due to its secondary structure it can be unambiguously aligned and there should also be sufficient characters that show some degree of variability to give reasonable resolution if data from closely related species are compared. Regardless of this, we were unable to find any correlation between s2m mutational patterns and host phylogenies. For instance, we were unable to assess whether the two copies of s2m found in the dog norovirus strains came from independent sources or if they are the result of some sort of duplication event and subsequent independent evolution (Figure 1B and Table 1), and the phylogeny of the deltacoronovirus s2m sequences was poorly resolved and did not match the phylogeny of the hosts (data not shown). There seems to be a number of loci that ‘permit’ certain substitutions and that these mutate quite quickly, masking any phylogenetic signal.

Although our analyses did not address the function of s2m *per se*, we believe that our observations might provide some clues as to how s2m might evolve and provide a selective advantage to the host viruses. We believe that s2m must have some sort of ‘autonomous’ function that does not require complex interactions with other parts of the viral genome/transcriptome as there do not appear to be any conserved flanking regions in s2m viruses (proximity to coding region, adjacent reading frames, nucleotide motifs etc.). Neither do there appear to be any conserved amino acid motifs in any of the annotated open reading frames except the GDD core of the RNA-dependent RNA polymerase when looking at protein sequence data from representatives from the four virus families (data not shown). The conserved nature of s2m might also imply that the s2m targets are homologous as all infected organisms are (relatively) closely related in an evolutionary perspective whereas the viruses are distantly related when looking both at molecular data and functionality (genome replication and transcription/translation strategies). An intracellular target also seems plausible, as all s2m containing viruses replicate in the cytoplasm and this is where s2m is likely to be available for interactions with the cellular machinery. The observation that s2m can apparently be transferred between unrelated viruses and remain functional (under selection pressure to maintain sequence and structure) also suggests strongly that the target for s2m is host-specific and not viral. Based on structural similarities between micro RNA (miRNA) hairpins involved in gene regulation, we propose that s2m functions through a RNA interference (RNAi)-like mechanism, possibly targeting homologous sequence loci in infected organisms. Recent observations using a reverse genetics-based approach and a recombinant Sindbis virus indicate that the required cellular machinery for this to function should be in place in human cells [19], and this model would also be consistent with an additive effect, where more copies of s2m would allow the formation of more miRNA/protein complexes, resulting in a more profound effect on target gene regulation.

Based on the finding of s2m in what appears to be newly emerging viruses, such as SARS-CoV, we also believe that s2m still maintains its mobility and will play a role in the future of virus evolution.

## Conclusions

The s2m sequence motif appears to be an active mobile genetic element that thus far can be found in four different families of + ssRNA viruses. It seems likely that s2m provides some kind of selective advantage for the viruses that contain the motif, and a possible function could be related to RNAi-like gene regulation of infected organisms.

## Methods

All sequence data were downloaded from GenBank. Only accessions where continuous sequence information from the 3’ end of the genome to the locus selected for phylogenetic analyses was available were used when investigating the evolutionary relationship between the (s2m-containing) viruses.

By using all the available sequence information from publications pertaining to s2m, several conserved sequences domains within s2m could be identified. These sequence motifs were used to perform nucleotide BLAST searches through the NCBI portal and >400 s2m-containing sequences could be identified. The s2m motifs (43–44 nucleotides long) were individually extracted and aligned, and the following consensus sequence could be generated: CGNGG(N)CCACGNNGNGT(N)ANNANCGAGGGT(N)ACAG (N’s in parenthesis indicate possible indels) for the conserved core region of s2m. This text string profile was used to search all viral sequences in GenBank using different combinations of the indicated indels and allowing for nucleotide substitutions.

All the alignments were constructed using the Clustal W algorithm [20] and manually edited using Bioedit (version 7.0.5.3; [21]). Only unambiguously aligned domains were included in the subsequent analyses. Phylogenetic analyses were performed using RAxML with the optimal GTR (General Time Reversible) with Gamma distribution model for amino acid substitution and 100 bootstrap replicates [22].

Minimally constrained trees were constructed to test whether trees with monophyly of s2m-containing sequences were significantly less likely than the optimal unconstrained tree. The primary constraint was to make all s2m-containing taxa monophyletic. If more than two s2m-containing clades were present alternate topologies were constructed in a pairwise manner to test topologies with two s2m clades constrained together. The optimal tree compatible with the constraint was calculated using RAxML with the above amino acid substitution model, followed by site likelihood calculation with RAxML. The Approximately Unbiased (AU) test was calculated using CONSEL [23].

## Competing interests

The authors declare that they have no competing interests.

## Authors’ contributions

TT conceptualized the project together with CMJ, collected the sequence data and constructed the amino acid alignments. TT also wrote the final version of the manuscript. TRB performed all the phylogenetic analyses and the AU testing. ABK did all the database mining and programming required to identify and tabulate the s2m-containing sequences in GenBank. All authors read and approved the final manuscript.

## Authors’ information

TT and ABK are senior staff scientists at the Norwegian Veterinary Institute (NVI; Section for virology and Section for epidemiology, respectively). TT works on emerging pathogens and ABK works on epidemiology and genetics of animal and fish pathogens. CMJ is section leader at NVI (Section for virology) and was the first to describe the s2m element in viruses. TRB works as a senior research scientist at the Institute for Marine and Environmental Technology and is currently investigating gene transfer and gene duplications in protists.

## Supplementary Material

Additional file 1 List of s2m-containing sequences found in Genbank. Click here for file

Additional file 2 **Figure S1, S2, S3 and S4.** For each virus family, the optimal tree with bootstrap support values is shown (identical to Figures 1, 2, 3 and 4). Each of the s2m-containing clades was numbered, from top to bottom without regard to taxonomy, and the total number of observed s2m gains was mapped onto the tree. Constraint trees were constructed to test a series of hypotheses for s2m gains. For example, all s2m-containing sequences were constrained to a single clade to see if a tree with only a single gain of the s2m sequence was significantly less likely given the alignment. Other less constrained trees were also constructed to test if trees with more than one s2m gain were also significantly less likely given the alignment. The specific constraints on s2m clades are shown in the table below. In all cases the differences in log likelihood and p-values for the different trees are shown in comparison to the most likely unconstrained tree. In addition to the Approximately Unbiased (AU) p-values, other test support values are also shown (np = bootstrap probablility, bp = bootstrap proportion, pp = posterior probability, kh = Kishino-Hasegawa, sh = Shimodaira-Hasegawa, wkh = weighted Kishino-Hasegawa, wsh = weighted Shimodaira-Hasegawa). Click here for file
