# Supplementary figures and images for: A mobile genetic element with unknown function found in distantly related viruses
Source: Virol J. 2013 Apr 25;10:132. doi: 10.1186/1743-422X-10-132 (PMC3653767; doi:10.1186/1743-422X-10-132)

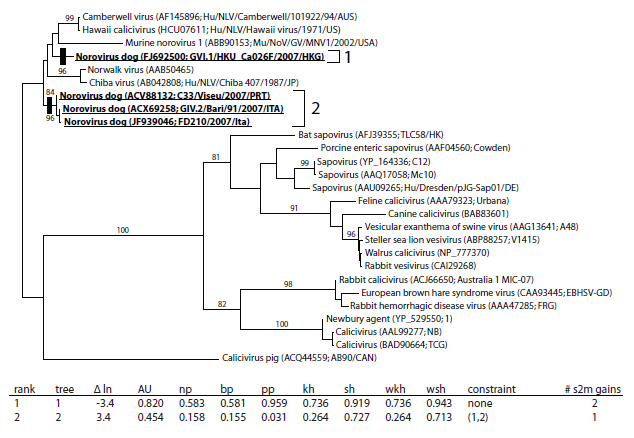


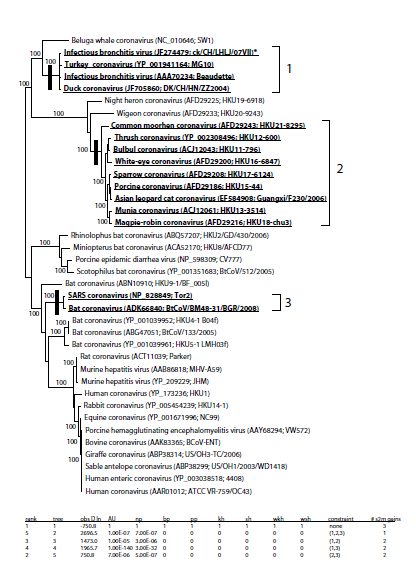


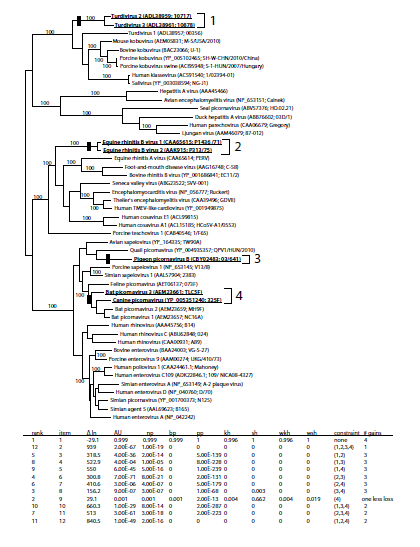


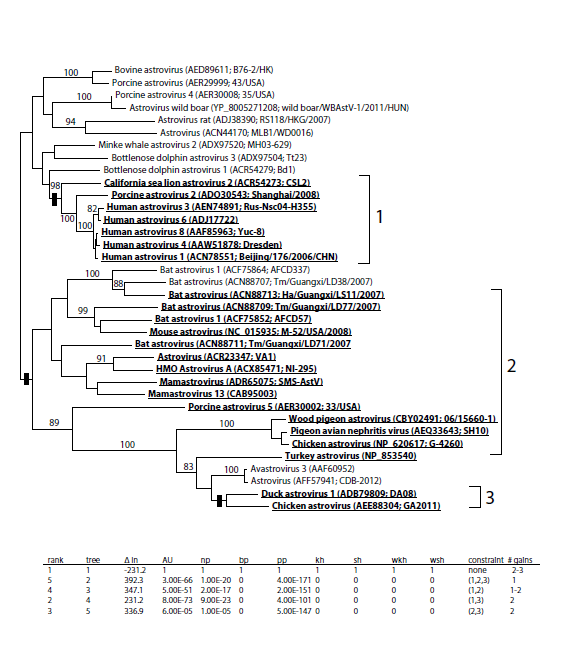

Supplement: Additional file 2 — Figure S1, S2, S3 and S4. For each virus family, the optimal tree with bootstrap support values is shown (identical to Figures 1, 2, 3 and 4). Each of the s2m-containing clades was numbered, from top to bottom without regard to taxonomy, and the total number of observed s2m gains was mapped onto the tree. Constraint trees were constructed to test a series of hypotheses for s2m gains. For example, all s2m-containing sequences were constrained to a single clade to see if a tree with only a single gain of the s2m sequence was significantly less likely given the alignment. Other less constrained trees were also constructed to test if trees with more than one s2m gain were also significantly less likely given the alignment. The specific constraints on s2m clades are shown in the table below. In all cases the differences in log likelihood and p-values for the different trees are shown in comparison to the most likely unconstrained tree. In addition to the Approximately Unbiased (AU) p-values, other test support values are also shown (np = bootstrap probablility, bp = bootstrap proportion, pp = posterior probability, kh = Kishino-Hasegawa, sh = Shimodaira-Hasegawa, wkh = weighted Kishino-Hasegawa, wsh = weighted Shimodaira-Hasegawa). [file 1743-422X-10-132-S2.doc]
